# Clinical significance of enlarged cardiophrenic lymph nodes by CT in advanced ovarian cancer

**DOI:** 10.3389/fonc.2023.1149139

**Published:** 2023-03-17

**Authors:** Sisi Song, Huizhu Chen, Gang Ning, Yingkun Guo, Xuesheng Li

**Affiliations:** ^1^ Department of Radiology, Key Laboratory of Birth Defects and Related Diseases of Women and Children of Ministry of Education, West China Second University Hospital, Sichuan University, Chengdu, China; ^2^ Department of Radiology, Deyang People’s Hospital, Deyang, Sichuan, China

**Keywords:** cardiophrenic lymph nodes, ovarian cancer, computed tomography, progression-free survival, clinical significance

## Abstract

**Aim:**

This study aims to assess the clinical influence of enlarged cardiophrenic lymph nodes (CPLN) on staging computed tomography (CT) among patients with advanced ovarian cancer.

**Methods:**

This retrospective cohort study included 320 patients with advanced epithelial ovarian cancer who underwent staging CT from May 2008 to January 2019. The CPLN diameter was the average of two radiologists’ measurements. Enlarged CPLN was defined as a short-axis diameter of ≥5 mm. Clinical and imaging findings, management decisions, and progression-free survival(PFS) were compared between patients with and without enlarged CPLN.

**Results:**

Enlarged CPLN was found in 129 (40.3%) patients, which was significantly associated with more pelvic peritoneal carcinomatosis (odds ratio [OR]: 6.61 with 95% confidence interval [CI]: 1.51–28.99), and involved the greater omentum (OR: 6.41, 95% CI: 3.05–13.46), spleen capsule nodules (OR: 2.83, 95% CI: 1.58–5.06), and liver capsule nodules (OR: 2.55, 95% CI: 1.57–4.17). The optimal cytoreduction rates did not differ between patients with and without enlarged CPLN (*p* = 0.656). The presence of enlarged CPLN had a significant negative influence on PFS (median PFS, 23.5 vs. 80.6 months, respectively, CPLN ≥5 mm versus <5 mm; *p* = 0.023) in patients with no RD after primary debulking surgery, but no adverse effect on PFS among patients with RD (median PFS, 28.0 vs. 24.4 months, respectively, CPLN ≥5 mm versus <5 mm; *p* = 0.359). However, enlarged CPLN on staging CT did not affect PFS in patients treated with neoadjuvant chemotherapy, with (median PFS, 22.4 vs. 23.6 months, respectively, CPLN ≥5 mm versus <5 mm; *p* = 0.360) or without RD (median PFS, 17.7 vs. 23.3 months, respectively, CPLN ≥5 mm versus <5 mm; *p* = 0.400). The enlarged CPLN showed a decreased trend in 81.6% (n = 80) of the patients with enlarged CPLN. No significant difference was found in PFS (*p* = 0.562) between patients with decreased and increased in the size of CPLN.

**Conclusions:**

Enlarged CPLN on staging CT is associated with more abdominal disease but is not reliable in predicting complete resection. Enlarged CPLN awareness is necessary for patients with a primary chance of complete resection of abdominal disease.

## Introduction

Ovarian cancer is the third most reported gynaecologic malignancy worldwide, with the highest mortality rate among gynaecological cancers (1). Most (>80%) of ovarian cancer is diagnosed at an advanced stage when the tumor has spread to the peritoneal cavity and upper abdominal organs, which is linked to the International Federation of Gynecology and Obstetrics (FIGO) stages III/IV and a lower overall survival (OS) rate (2). Patients who are suspected of ovarian cancer most commonly undergo a staging computed tomography (CT) scan to evaluate the extent of the disease, allocate for treatment, and provide a baseline for treatment response assessment (3).

Some patients who are eligible for primary debulking surgery (PDS), whereas others who are not suitable for PDS are treated with neoadjuvant chemotherapy (NAC) followed by interval debulking surgery. Cardiophrenic lymph nodes (CPLN) are frequently visible on CT imaging of patients with advanced ovarian cancer, with detection rates ranging from 11% to 62%, depending on the utilized selective diameter and the investigated patient cohort (4–8). Debulking surgery for advanced ovarian cancer does not routinely include opening the thorax (9). Even systematic lymphadenectomy does not commonly extend to lymph nodes above the diaphragm (9). Thus, the clinical significance of CPLN is unclear, causing difficulty in the decision-making process.

In terms of the clinical influence of radiologically enlarged CPLN in ovarian cancer, several studies (5, 10) revealed that enlarged CPLN is related to impaired progression-free survival (PFS) and OS in patients with macroscopically complete tumor resection after PDS; however, enlarged CPLN have no effect on survival in patients with postoperative residual tumor. Mert et al. (6) support the aforementioned results by characterizing abnormal CPLN using three different definitions, whereas Oommen et al. (11) demonstrated that enlarged CPLN did not adversely affect OS, but adversely affected PFS. However, these studies only discussed whether CPLN was correlated with PFS and OS in different populations and did not compare whether CPLN had different effects on PFS and OS between people who underwent PDS and those who underwent NAC.

This study aimed to gather further evidence regarding the clinical significance of enlarged CPLN on staging CT by examining the differences in the disease burden and treatment outcomes among patients with advanced epithelial ovarian cancer and comparing the different effects of enlarged CPLN on PFS between people who underwent PDS and those who underwent NAC.As a secondary outcome, we evaluated the impact of CPLN on oncological outcome, i.e.,whether enlarged CPLN had a predictive value for abdominal disease or complete tumor resection.

## Methods

### Study population

**Figure 1:**
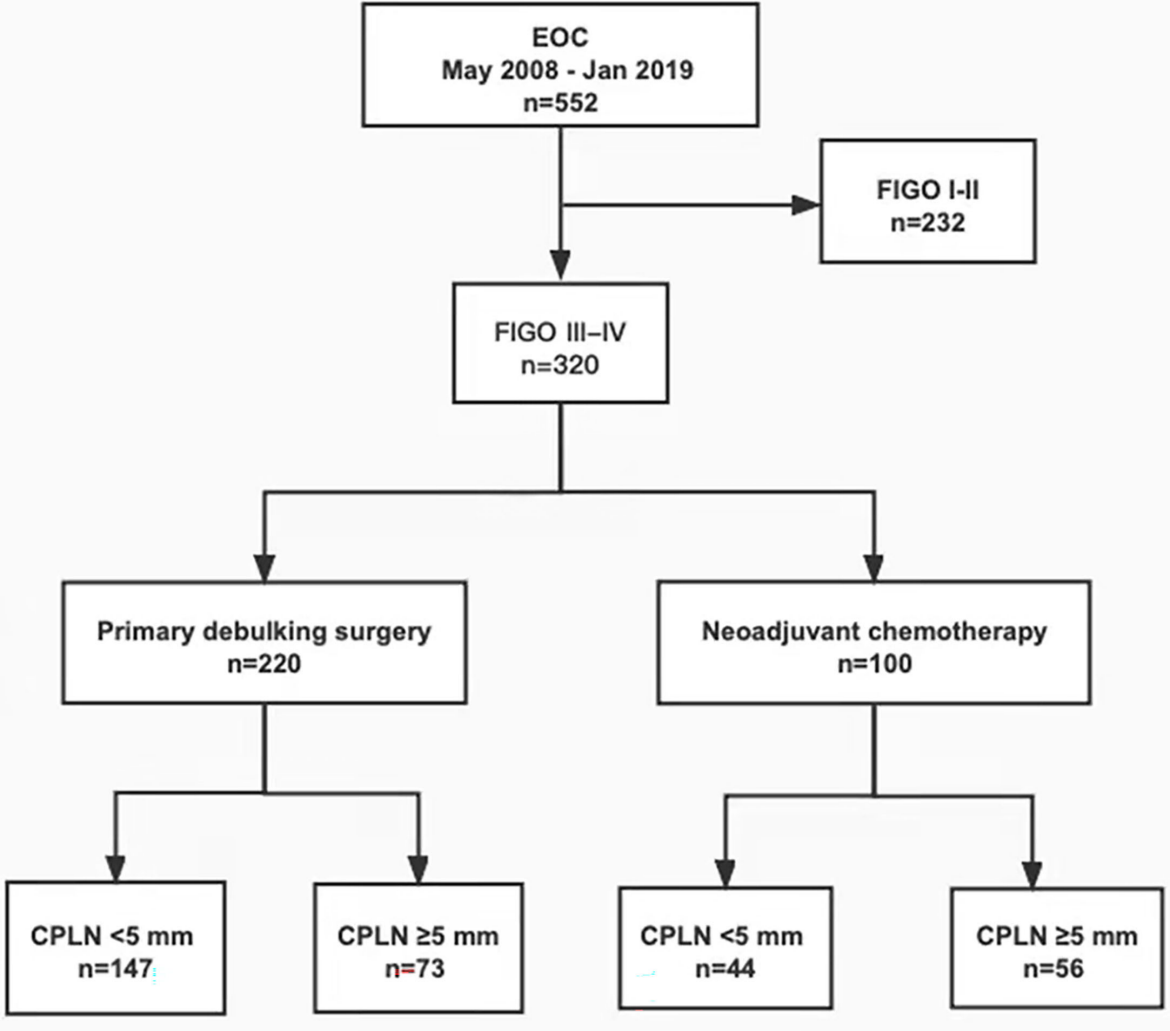
Flowchart showing patient flow of the patient population involved in the study. EOC, epithelial ovarian cancer; FIGO, International Federation of Gynecology and Obstetrics; CPLN, cardiophrenic lymph nodes.

This single-centre retrospective cohort study included 320 patients diagnosed with stage III or IV primary epithelial ovarian cancer from May 2008 to January 2019 in our hospital, according to the FIGO. The statement of the involved patient population in the study is depicted in the flowchart in **Figure 1**. All patients had staging CT scans with intravenous contrast before therapy. Patients who did not have staging CT scans in our institution or were treated in part with surgery or chemotherapy elsewhere were excluded from the study. On the basis of clinical assessment of the extent of their disease and performance status, some patients were eligible for PDS followed by adjuvant chemotherapy (n = 220, 68.8%), and patients not suitable for primary surgery were treated with NAC followed by interval debulking surgery (n = 100, 31.2%). Clinicopathological parameters, such as age, tumor markers, treatment history, and histologic grades, were collected and analyzed for those patients. No residual disease (NRD) refers to the absence of a macroscopically visible intra-abdominal tumor, which is only equivalent to microscopic residual disease (RD). Optimal cytoreductive surgery is defined as RD of <1 cm in maximum diameter or thickness (12). This study was approved by the Institutional Review Board (IRB) of West China Second University Hospital; the IRB reference number was 2020173. All patient-sensitive information was kept strictly confidential and used only for this study.

### CT technique

Abdominal staging CT scans were performed in one of the following multidetector CT scanners using standard scan parameters (13) Brilliance 6-Slice CT Scanner (Philips, Best, The Netherlands) and NeuViz 128 CT Scanner (Neusoft Medical Systems, China). CT scan was performed from the top of the diaphragm to the lower pubic symphysis using the following parameters: 120 kVp, 100–320 mA, 0.9 s of the rotation time, 512 × 512 matrices, 2 mm of reconstruction thickness, and 1 mm of reconstruction interval. Images in arterial and venous phases were obtained following the injection of 80–100 mL of contrast material (Iohexol, GE Healthcare, USA) at a rate of 2.5–3.5 mL/s using an automatic power injector. The arterial phase images were taken after a delay of 20–25 s, whereas the venous phase images were obtained after a delay of 60–70 s.

### Image analysis

All CT images were independently reviewed visually by two blinded radiologists, with 7 and 9 years of experience. CPLN was considered present when there were soft tissue density foci that appeared and disappeared in the cardiophrenic spaces while scrolling through images in axial planes (11). The CPLN diameter was the average of both radiologists’ measurements. CPLN with a short-axis diameter of ≥5 mm along the short axis was regarded as radiologically suspicious, according to the policy of the European Society of Urogenital Radiology (ESUR) guidelines (4). This study neither considered the presence of abnormal CPLN on staging CT scan as a criterion for stage IV disease nor used CPLN to define the RD. Furthermore, ascites, para-aortic lymph nodes, pelvic lymph nodes, and the localisation of intra-abdominal carcinomatosis were assessed. Enlarge para-aortic lymph nodes and pelvic lymph nodes were defined as short diameters ≥10 mm (4).

### Statistical analysis

All analyses were performed using IBM Statistical Package for the Social Sciences version 26.0. Comparison of baseline patient and tumor characteristics between patients with and without abnormal CPLN was evaluated using the Mann–Whitney U test for age and preoperative cancer antigen 125 (CA-125), as well as the chi-squared test for categorical variables. PFS was calculated using the Kaplan–Meier method and compared between groups using the log-rank test. All calculated *p*-values were two sided, and *p*-values of <0.05 were considered statistically significant.

## Results

### Clinicopathological parameters

From May 2008 to January 2019, 320 patients underwent surgery and were diagnosed with primary advanced ovarian cancer at our institution. Of these, 220 (68.8%) patients had PDS followed by adjuvant chemotherapy and 100 (31.2%) had NAC followed by interval debulking surgery. There were 234 (73.1%) patients with stage IIIC disease, whereas the majority (89.4%, n = 286) has high-grade serous histology. **Table 1** compares the characteristics of patients with and without enlarged CPLN. No difference was found in age, FIGO grade, histology, or RD between the groups with and without abnormal CPLN (all, *p* > 0.05). However, enlarged CPLN were associated with a higher preoperative CA-125 level (median value: 1508.0 U/mL vs. 498.0 U/mL; *p* = 0.000) and a higher rate of ascites (96.9% vs. 88.0%; *p* = 0.005).

**Table 1:** Patient and tumor characteristics by presence of enlarged CPLN where enlarged CPLN defined as ≥ 5 mm on the short axis.

Characteristic	Total (N = 320)	Normal CPLN (N = 191)	Enlarged CPLN (N = 129)	*p*-Value
Age (years), median (IQR)	50.0 (44.3–57.0)	50.0 (45.0–57.0)	50.0 (44.0–56.0)	0.551
FIGO stage, N (%)				0.170
IIIA	28 (8.8%)	21 (11.0%)	7 (5.4%)	
IIIB	42 (13.1%)	27 (14.1%)	15 (11.6%)	
IIIC	234 (73.1%)	136 (71.2%)	98 (76.0%)	
IV	16 (5%)	7 (3.7%)	9 (7.0%)	
Histology, N (%)				0.317
High-grade serous	286 (89.4%)	168 (88.0%)	118 (91.5%)	
others	34 (10.6%)	23 (12.0%)	11 (8.5%)	
Serum CA-125 (U/mL), median (IQR)	562.8 (298.8–2245.4)	498.0 (193.1–1438.8)	1508 (483.7–3303.7)	0.000
Ascites, N (%)	293 (91.6%)	168 (88.0%)	125 (96.9%)	0.005
Residual disease, N (%)				0.383
No residual disease	148 (46.3%)	91 (47.6%)	57 (44.2%)	
0–1 cm	105 (32.8%)	57 (29.8%)	48 (37.2%)	
>1 cm	51 (15.9%)	31 (16.2%)	20 (15.5%)	
unknown	16 (5.0%)	12 (6.3%)	4 (3.1%)	

CPLN, cardiophrenic lymph node; IQR, interquartile range; FIGO, International Federation of Gynecology and Obstetrics; CA-125, cancer antigen 125.

### Radiological detection of CPLN

**Figure 2:**
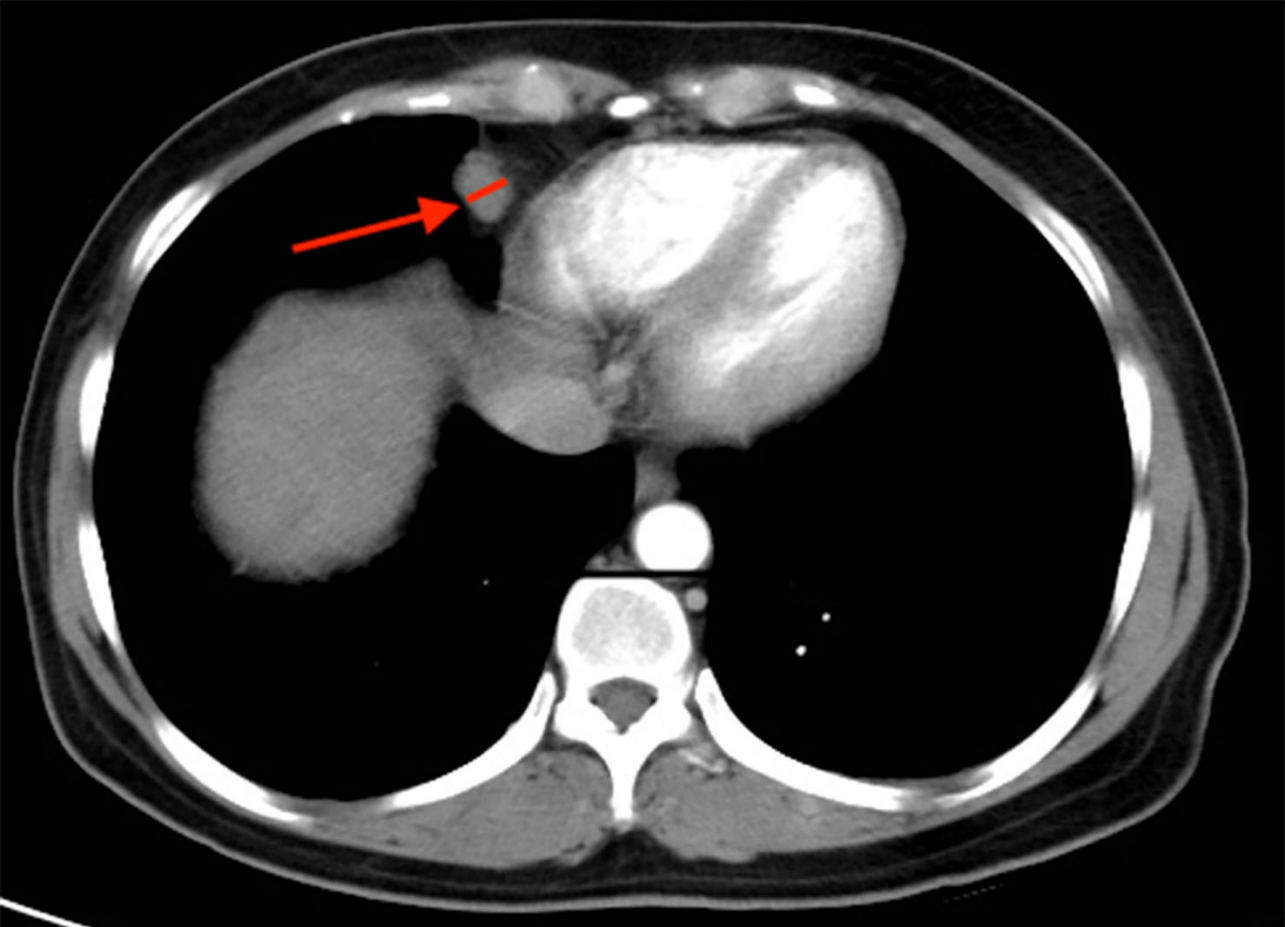
Staging CT image showing an example of cardiophrenic lymph node (CPLN) of 9.4 mm in the short axis and located in the anterior right location.

Staging CT scans revealed CPLN of any size in 72.2% (n = 231) of the 320 patients, and 40.3% (n = 129) of patients had lymph nodes of ≥5 mm on the short axis, as shown in **Figure 2**. The enlarged CPLN had a mean short axis diameter of 7.3 ± 2.2 mm with a range of 5–15.9 mm and a mean long axis diameter of 11.7 ± 4.1 mm with a range of 5.9–21.5 mm. A mean of 1.8 ± 1.0 enlarged CPLN with a range of one to five nodes was observed among patients with enlarged CPLN. Most of the patients (n = 120, 93.0%) had enlarged nodes in the anterior right location. Thirty-one (24.0%) and 0 patients had enlarged nodes in the anterior left and posterior location, respectively. The majority (79.8%, n = 103) of enlarged CPLN had smooth margins, whereas 20.1% (n = 26) had lobulated or irregular margins. Most of the enlarged CPLN (93.8%, n = 121) had homogeneous attenuation. Only five and three patients showed nodes with a low-density centre and calcification, respectively.

### Predictive value of enlarged CPLN for abdominal disease

Radiologically, 93.8% (n = 300) of all patients had pelvic peritoneal carcinomatosis. The greater omentum was involved in 249 (77.8%) patients, and 96 (30.0%) and 59 (18.4%) patients had suspicious lesions on the liver and spleen surface, respectively. **Table 2** compares the radiological findings of patients with and without enlarged CPLN. Here, we discovered a significant association between the presence of enlarged CPLN and the following radiological abdominal disease, as presented in order of magnitude of effect: pelvic peritoneal carcinomatosis (odds ratio [OR]: 6.61 with 95% confidence interval [CI]: 1.51–28.99), greater omentum involved (OR: 6.41, 95% CI: 3.05–13.46), spleen capsule nodules (OR: 2.83, 95% CI: 1.58–5.06), and liver capsule nodules (OR: 2.55, 95% CI: 1.57–4.17). However, the association between positive pelvic lymph nodes and enlarged CPLN was weaker (OR: 1.96, 95% CI: 1.03–3.71). We found no link between para-aortic lymph node or mesentery involvement and CPLN(*p* > 0.05).

**Table 2:** Radiological parameters in patients’ abdominal disease with and without enlarged CPLN where enlarged CPLN defined as ≥ 5 mm on the short axis.

Characteristic, N (%)	Total (N = 320)	Normal CPLN (N = 191)	Enlarged CPLN (N = 129)	*p*-Value
Liver capsule nodules	96 (30.0%)	42 (22.0%)	54 (41.9%)	0.000
Spleen capsule nodules	59 (18.4%)	23 (12.0%)	36 (27.9%)	0.000
Greater omentum involved	249 (77.8%)	129 (67.5%)	120 (93.0%)	0.000
Mesentery involvement	11 (3.4%)	5 (2.6%)	6 (4.7%)	0.361
Pelvic peritoneal carcinomatosis	300 (93.8%)	173 (90.6%)	127 (98.4%)	0.004
Para-aortic lymph nodes	98 (30.6%)	59 (30.9%)	39 (30.2%)	0.900
Pelvic lymph nodes	44 (13.8%)	20 (10.5%)	24 (18.6%)	0.038

CPLN, cardiophrenic lymph node.

### Predictive value of enlarged CPLN for complete tumor resection

Our cohort of 220 patients underwent PDS followed by adjuvant chemotherapy, whereas the remaining 100 underwent NAC followed by interval debulking surgery. PDS was performed more on patients without enlarged CPLN (n = 147, 45.9%) than on those with enlarged CPLN (n = 73, 22.8%) (*p* = 0.000). Conversely, a significantly higher proportion of patients with enlarged CPLN underwent NAC (n = 56, 17.5%) than those without enlarged CPLN (n = 44, 13.8%) (*p* = 0.000). The overall rate of optimal cytoreductive surgery was 83.3%, with the patients underwent NAC (93.9%) having a higher rate than those underwent PDS (78.3%) (*p* = 0.000). No difference was found in the rates of optimal cytoreduction between patients with and without enlarged CPLN (*p* = 0.656), regardless of PDS (*p* = 0.938) or NAC (*p* = 0.587).

### Prognostic impact of enlarged CPLN

**Figure 3:**
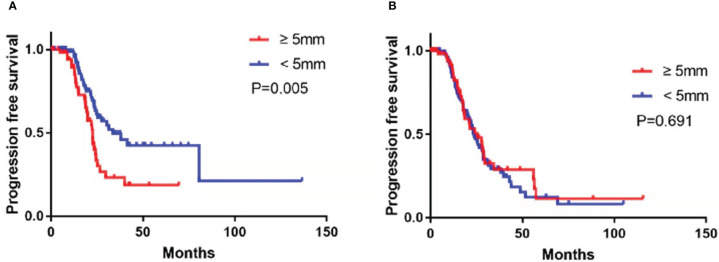
Progression-free survival in patients with no residual disease **(A)** and residual disease **(B)** after primary debulking surgery or neoadjuvant chemotherapy with cardiophrenic lymph nodes (CPLN) of ≥5 mm versus <5 mm.

Follow-up data were available for 286 (89.4%) patients, and the remaining 34 (10.6%) were lost to follow-up. The cohort’s median follow-up time was 36.3 months (IQR: 27.5–45.1). There were 150 (52.4%) patients with RD and 136 (47.6%) with NRD, with recurrence occurring in 50.3% (n = 144). Enlarged CPLN was determined in 55 (40.4%) patients in the NRD group. The presence of enlarged CPLN in the NRD group had a significant negative influence on PFS, with a log-rank *p*-value of 0.005 (**Figure 3A**). And the median PFS was 22.6 months for patients with enlarged CPLN, compared with 33.7 months for patients without enlarged CPLN, respectively. In the RD group, No significant adverse effect was observed on PFS among the patients with or without enlarged CPLN (median PFS, 25.4 vs. 23.7 months, respectively; *p* = 0.691; **Figure 3B**).

**Figure 4:**
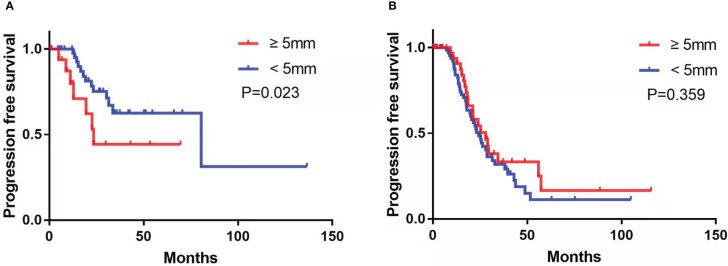
Progression-free survival in patients with no residual disease **(A)** and residual disease **(B)** after primary debulking surgery with cardiophrenic lymph nodes (CPLN) of ≥5 mm versus <5 mm.

**Figure 5:**
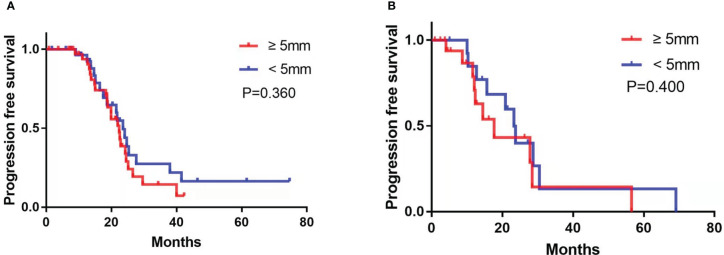
Progression-free survival in patients with no residual disease **(A)** and residual disease **(B)** after neoadjuvant chemotherapy with cardiophrenic lymph nodes (CPLN) of ≥5 mm versus <5 mm.

Subgroup analysis revealed that among the patients treated with PDS, the presence of enlarged CPLN on staging CT had a significant negative influence on PFS in the NRD group (median PFS, 23.5 vs. 80.6 months, respectively, CPLN ≥5 mm versus <5 mm; *p* = 0.023; **Figure 4A**), but no significant adverse effect on PFS in the RD group (median PFS, 28.0 vs. 24.4 months, respectively, CPLN ≥5 mm versus <5 mm; *p* = 0.359; **Figure 4B**). However, enlarged CPLN on staging CT did not affect the PFS in patients treated with NAC in either NRD (median PFS, 22.4 vs. 23.6 months, respectively, CPLN ≥5 mm versus <5 mm; *p* = 0.360; **Figure 5A**) or RD groups (median PFS, 17.7 vs. 23.3 months, respectively, CPLN ≥5 mm versus <5 mm; *p* = 0.400; **Figure 5B**).

### Follow-up of enlarged CPLN

**Figure 6:**
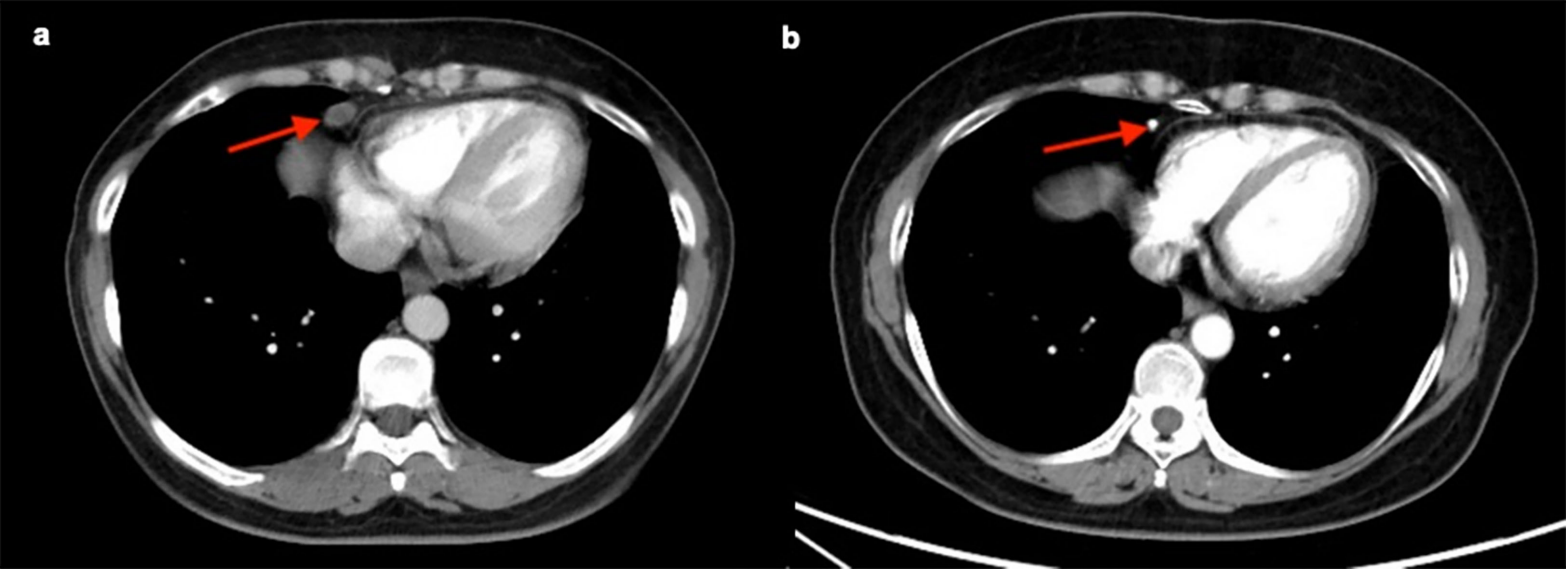
Staging CT image of a patient with FIGO stage IIIC ovarian cancer who presented with an enlarged cardiophrenic lymph node (CPLN) of 9.7 mm in the short axis **(A)**. The CPLN had decreased with a short axis diameter of 3.0 mm 4 years later, and the patient had no recurrence **(B)**.

**Figure 7:**
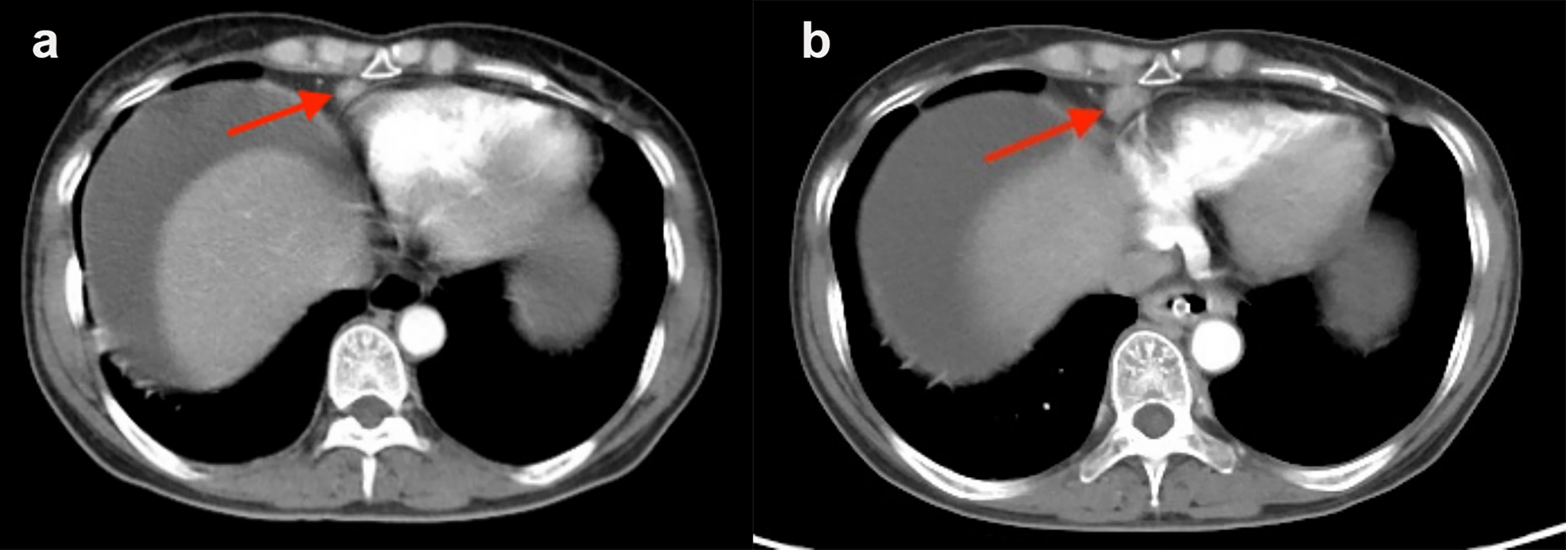
Staging CT image of a patient with FIGO stage IIIC ovarian cancer who presented with an enlarged cardiophrenic lymph node (CPLN) of 6.4 mm in the short axis **(A)**. The CPLN had grown to 11.3 mm 5 months later, but the patient had no recurrence in the abdomen **(B)**.

**Figure 8:**
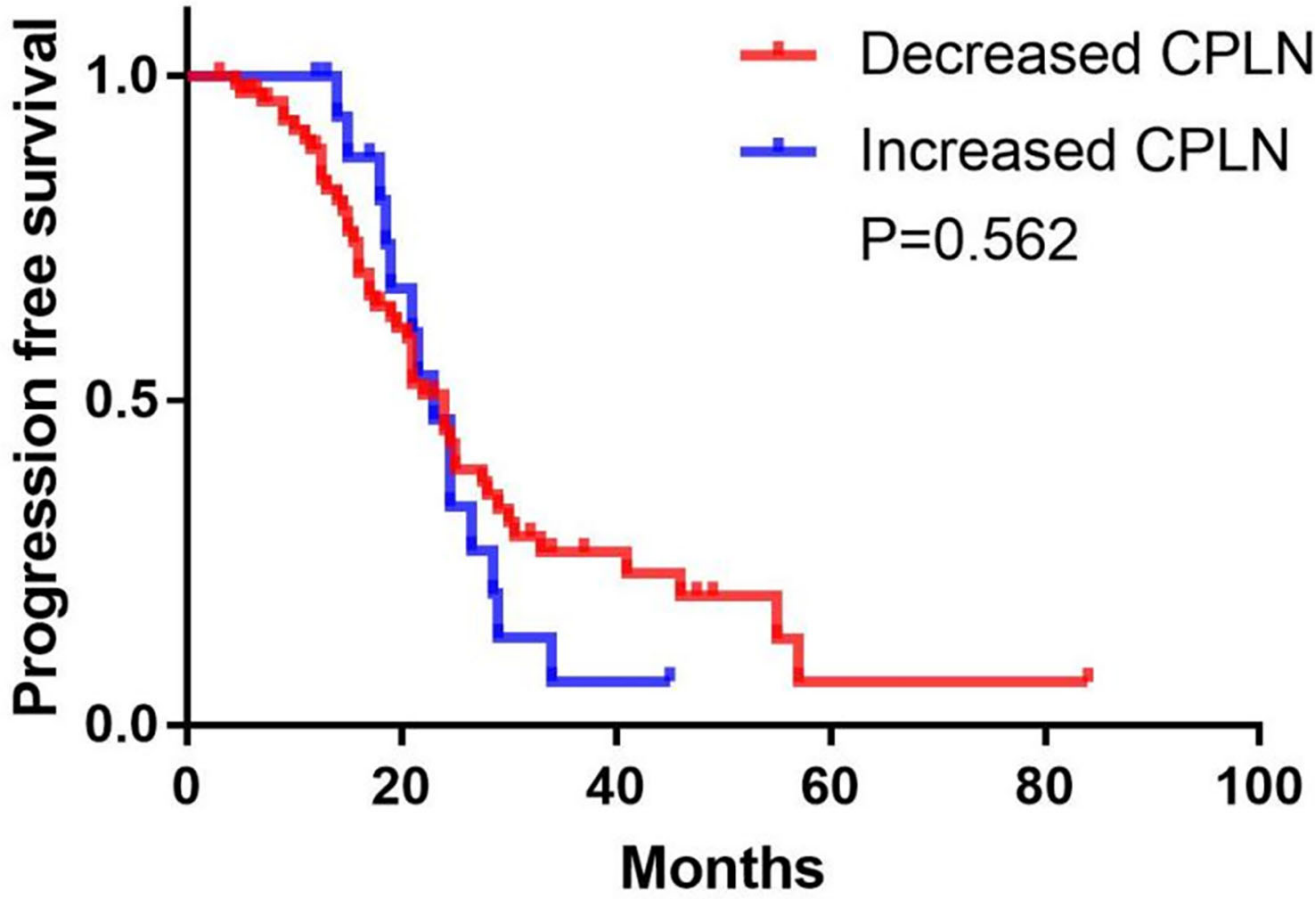
Progression-free survival in patients with increased versus decreased in the size of cardiophrenic lymph nodes (CPLN).

A follow-up CT scan was performed in 98 (76.0%) patients in the enlarged CPLN group, wherein 62.2% (n = 61) had recurrence, with a median PFS of 24.0 (95% CI: 21.7–26.3) months. The size of the CPLN show a decreased trend in 81.6% (n = 80) of patients (**Figure 6**), whereas 18 (18.4%) patients had larger CPLN after therapy than that at staging CT (**Figure 7**). No significant difference was found in PFS (*p* = 0.562) between patients with decreased and increased in size of CPLN (**Figure 8**).

## Discussion

We studied the clinical characteristics and treatment responses of patients with advanced ovarian cancer with detected enlarged CPLN *via* CT in developing countries. Enlarged CPLN was associated with more abdominal disease but was not reliable to predict complete resection. Furthermore, abnormal CPLN exhibited a trend towards poorer PFS in patients with advanced ovarian cancer with NRD in the PDS group. However, this effect was not observed in individuals with RD in the PDS or NAC group.

CPLN are frequently visible on CT imaging of patients with advanced ovarian cancer. Our cohort analysis revealed that 72.2% of patients had CPLN of any size on staging CT scans and 40.3% had lymph nodes measuring ≥5 mm in the short axis. Furthermore, the literature reports detection rates ranging from 11% to 62%, depending on the utilized selective diameter and the investigated patient cohort (4–8). On an individual patient basis, determining whether CPLN shown on CT reflects stage IV disease or is related to concomitant infective or inflammatory diseases is difficult (11). The optimal cutoff size for pathologic CPLN is unknown. FIGO stage shift is heavily dependent on the used radiological short-axis cutoff. Collectively, these raise the question of whether a cancer-affected CPLN has clinical significance, or whether the current FIGO classification is imprecise in defining FIGO stage IVB disease with clinical relevance. A similar controversy about the significance of abdominal wall metastases (14) and inguinal nodes (15) has erupted. Several studies demonstrate no difference in results when a cutoff of 5 mm is used (7, 16), whereas other studies use 7 (17) or 10 mm (9). Therefore, we choose a 5 mm threshold to define enlarged CPLN in line with the ESUR guidelines (4). Unfortunately, we did not pathologically confirm the involved abnormal nodes because we did not routinely remove CPLN. Relevant literature reported the percentage of confirmed enlarged CPLN using histology ranges from 85% to 95% (5, 9, 17, 18).

Our data revealed a stronger association between enlarged CPLN and peritoneal carcinomatosis than between enlarged CPLN and lymph node status of the typical regional nodes in the pelvic and para-aortic regions. This finding indicates that the CPLN might not be just a continuum of the para-aortic lymphatic drain. CPLN may simply be a sign of extensive peritoneal carcinomatosis (5–8).

Several studies (5–8) demonstrated an inverse impact of enlarged CPLN on complete resection rates. Luger et al. (10) applied a new score to predict complete tumor debulking during upfront surgery by combining the radiological CPLN status, the volume of ascites, and CA-125 level at baseline. This score application resulted in a substantial increase of the negative predictive value to 87% (10). However, our study revealed no difference in the optimal cytoreduction rates among all patients with and without enlarged CPLN, which is congruent with that of Oommen et al. (11). The complete resection rate varies depending not only on the abdominal lesions but also on the level of surgery in different regions, which explains the different study findings. The enlarged CPLN seen on staging CT in patients with advanced ovarian cancer is most likely due to direct disease spread from the peritoneum, which could affect treatment and prognosis. However, predicting complete resection based on the enlarged CPLN in imaging is not reliable.

Several studies revealed that enlarged CPLN is related to poor patient outcomes (5, 6, 8, 16, 19). Most of the studies (5, 6, 10) revealed that enlarged CPLN is related to impaired PFS and OS in patients with macroscopically completely resected tumors after PDS. Oommen et al. (11) demonstrated that enlarged CPLN did not adversely affect OS but adversely affected PFS. This observation is supported by our findings, which show a significant negative influence on PFS in patients with advanced ovarian cancer with radiologically detected enlarged CPLN and complete intra-abdominal tumor excision. However, we did not analyse the OS because of a few deaths in our cohort. Additionally, we found a significant negative influence of the presence of enlarged CPLN on staging CT on DFS with RD in the PDS group, but without significant adverse effect on DFS among the patients with RD. In contrast, enlarged CPLN on staging CT did not affect PFS in patients treated with NAC with either NRD or RD. Hence, for patients with a primary chance of complete resection of abdominal disease, enlarged CPLN awareness is necessary, whereas for the rest of the patients, we need not to focus on enlarged CPLN.Moreover, recently, the clinical significance of metastatic disease on hepatoceliac lymph node and mesenteric lymph node are under investigation, as in other anatomical station. Gallotta et al. (20, 21) demonstrated that metastatic hepatoceliac lymph node was a marker of disease severity associated with the worst oncological outcome and that mesenteric lymph node was associated with a high rate of isolated aortic and celiac trunk lymph node recurrence.

Little is known regarding the benefit of lymphadenectomy outside of the abdominal cavity in patients with advanced ovarian cancer. The Lymphadenectomy in Ovarian Neoplasms trial was designed to evaluate pelvic and para-aortic lymphadenectomy in advanced ovarian cancer after the success of intra-abdominal complete debulking and revealed no survival benefit with systemic lymphadenectomy (22). Prader et al. (5) recently found that patients who underwent radiologically enlarged CPLN removal did not confer a significant survival benefit compared with a matched control population who did not undergo enlarged CPLN removal. Similarly, Lopes et al. (23) completed transdiaphragmatic excision on 24 patients with cardiophrenic node involvement on staging CT and revealed that the surgery did not provide a significant survival advantage, but it did aid in attaining complete cytoreduction and verifying the presence of extra-abdominal illness. At our institution, preoperative radiography examination of enlarged CPLN has not been routinely incorporated into ovarian cancer staging and treatment considerations for technical and other reasons. Therefore, we need a larger multicenter study to determine the influence of CPLN excision in patients with advanced ovarian cancer.

Our study has a few limitations that are commonly associated with its retrospective nature, but we performed a central two-reader re-review of all CT scans to overcome it. Furthermore, a larger multicentric series should confirm the analysis results. Moreover, the number of patients included in the final analysis was limited. Hence, we did not analyse the OS because of a few deaths in our cohort. Last, we did not routinely remove CPLN during the years of study, so we did not pathologically verify the involved abnormal nodes and instead relied on existing studies for cutoff values.

## Conclusions

In conclusion, enlarged CPLN on staging CT in patients with advanced ovarian cancer is associated with more abdominal disease but is not reliable in predicting complete resection based on the expanded CPLN in imaging. Enlarged CPLN awareness is necessary for patients who have a primary chance of complete resection of abdominal disease because of their poorer survival compared with patients without enlarged CPLN. The majority of enlarged CPLN show a decreasing trend, without a significant difference in PFS between patients with decreased and increased in the size of CPLN.

## Data availability statement

The raw data supporting the conclusions of this article will be made available by the authors, without undue reservation.

## Ethics statement

The studies involving human participants were reviewed and approved by the Institutional Review Board (IRB) of West China Second University Hospital (2020173). The patients/participants provided their written informed consent to participate in this study.

## Author contributions

SS, HC,GN, XL and YG designed this study, SS, HC and XL collected data, SS, HC analyzed the data, SS wrote the manuscript text. All authors contributed to the article and approved the submitted version.
